# Diagnosis and treatment of gastrointestinal dysfunction in hereditary TTR amyloidosis

**DOI:** 10.1007/s10286-019-00628-6

**Published:** 2019-08-26

**Authors:** Laura Obici, Ole B. Suhr

**Affiliations:** 1 Amyloidosis Research and Treatment Centre, Fondazione IRCCS Policlinico San Matteo, Viale Golgi, 19, 27100 Pavia, Italy; 2 Department of Medicine, Public Health and Clinical Medicine, Umeå University, Umeå, Sweden

**Keywords:** Amyloidosis, Transthyretin, Gastroparesis, Diarrhoea, Malabsorption

## Abstract

**Purpose:**

To review the management of gastrointestinal symptoms in patients with hereditary transthyretin amyloidosis, discussing diagnostic evaluations, assessment of disease progression and therapeutic strategies that could be implemented in routine practice.

**Methods:**

Literature review. Key search terms included “gastrointestinal symptoms”, “autonomic neuropathy”, “hereditary transthyretin amyloidosis” and “familial amyloid polyneuropathy”.

**Results:**

Gastrointestinal disturbances are a common and serious manifestation of hereditary transthyretin amyloidosis, with significant effects on patients' quality of life and demonstrating a strong association with mortality. Gastrointestinal involvement is more often subclinical in the early stages of the disease, although in some patients gastric and/or bowel abnormalities may be the inaugural symptoms. In both cases, under-recognition, delayed investigation and suboptimal treatment frequently occur. A clear understanding of the mechanisms underlying gastrointestinal dysfunction in hereditary transthyretin amyloidosis is still lacking, but similar to diabetic enteropathy, multiple pathophysiological alterations seem to play a role.

**Conclusions:**

Early detection and treatment of gastrointestinal disturbances is key to the successful treatment of this devastating disease. Gastroenterologists play a valuable role in both the diagnosis and the timely management of gastrointestinal symptoms in hereditary transthyretin amyloidosis and should, therefore, be part of a multidisciplinary and comprehensive approach to this disorder.

## Introduction

Several manifestations of hereditary transthyretin amyloidosis (ATTRv) are related to the early and progressive damage to the autonomic nervous system. Similar to diabetic autonomic neuropathy, impairment of the enteric nervous system and its extrinsic efferent sympathetic and parasympathetic innervation results in altered motility and defective secretory function within the entire gastrointestinal (GI) tract [1]. Patients complain of a large spectrum of disabling symptoms that are mostly attributable to reduced upper and lower GI motor function. Development and maintenance of early satiety, postprandial fullness, bloating, nausea, vomiting and weight loss are ascribed to impaired gastric empting, although the correlation between objective measures and reported symptoms is not clear [2, 3]. Constipation, alternating constipation and diarrhoea, chronic diarrhoea, abdominal pain and faecal incontinence are associated with abnormal bowel function and contribute significantly to the development of malnutrition that characterizes the later stages of the disease [4].

The prevalence of GI manifestations in ATTRv has been investigated extensively across different genotypes and phenotypes. In a cross-sectional study from the Transthyretin Amyloidosis Outcomes Survey (THAOS) registry, between 56 and 69% of patients, depending on mutation, reported GI disturbances [5]. Symptoms are more frequent in V30M than in non-V30M patients and significantly more common in the early onset than in the late-onset phenotype. These findings are fully consistent with previous observations from other series [6–8]. The frequency and type of GI manifestations in ATTRv patients from THAOS are displayed in Table 1. Unintentional weight loss is the most common, followed by early satiety and symptoms from the lower GI tract. A considerable number of patients complain of faecal incontinence, a devastating condition with regard to the patient's quality of life. Between 10 and 20% experience nausea and vomiting. Notably, patients may report weight loss before they develop any symptoms from the GI tract and even before symptoms of neuropathy occur [9]. GI bleeding is rarely if ever caused by ATTRv. Likewise, intestinal obstruction is not an expected complication. Patients with such symptoms should therefore be investigated as non-ATTR amyloidosis [10].

**Table 1:** Frequency of gastrointestinal symptoms reported by patients with hereditary transthyretin amyloidosis at the time of enrolment in the THAOS registry (Adapted from [5])

Symptom	Frequency (%)
Unintentional weight loss	31.5
Early satiety	26.4
Diarrhoea/constipation	24.3
Constipation	20.9
Diarrhoea	19.8
Nausea	17.1
Vomiting	13.4
Faecal incontinence	6.2

Gastrointestinal manifestations result in higher disease burden and dramatically affect patients' quality of life, limiting working and social activities, increasing hospitalizations and causing significant psychological distress in family and caregivers as well. In the THAOS study, both upper and lower GI symptoms were negatively associated with the EuroQol EQ-5D index score, with faecal incontinence having the most profound effect [5]. The incidence of GI disturbances rises over time and has a negative impact on nutritional status and prognosis [11]. Malnutrition measured by the modified body mass index (mBMI), where BMI is multiplied by serum albumin (g/L) to compensate for oedema, is strongly correlated with patient survival and with the outcome after liver transplantation [12, 13]. Therefore, GI involvement has a substantial impact on treatment choices and confers a heightened risk of mortality [14].

As in diabetic enteropathy, GI involvement in ATTRv may be subclinical in the early stages of the disease, resulting in under-recognition, delayed investigation and suboptimal treatment. On the other hand, isolated bowel abnormalities may be the presenting symptoms in some patients [15]. Not infrequently, these patients undergo several rounds of endoscopic evaluations before the diagnosis is ultimately established. As ATTR amyloid deposition is mostly submucosal, endoscopy does not show significant findings, and biopsies are often not taken. Different studies have proven that deep sampling, including submucosal vessels, is required to detect ATTRv amyloid deposits [16, 17]. A higher amyloid content has been reported in the upper GI tract compared with the lower, so if care is taken to obtain sufficiently deep biopsies, the stomach and the duodenum are probably the most suitable sites from which informative samples can be obtained [17, 18].

In this review, we discuss diagnostic investigations and therapeutic strategies for GI dysfunction in ATTRv. To highlight the most relevant problems encountered in the daily management of GI manifestations in ATTRv we present and comment on two cases that provide useful real-life information.

## Case 1: a woman with recurrent vomiting and peripheral neuropathy

A 69-year-old woman presented with progressive numbness in hands and feet, abnormal gait and evidence, on nerve conduction studies, of a moderate, mixed, predominantly sensory polyneuropathy at limbs. She was previously extensively evaluated and also treated with intravenous immunoglobulins without benefit. In her medical history she reported a 20-kg weight loss over the past 3 years, associated with recurrent episodes of vomiting lasting 3–5 days and requiring frequent hospitalizations. Gastroenterological evaluations included a double-contrast barium swallow, a total-body PET and two gastroscopy procedures performed 1 year apart. No significant findings were detected and biopsies were not taken. She was started on antidepressants. On evaluation, the patient was ambulatory but unable to walk on heels and toes. Neurological examination showed reduced thermal, pinprick and light-touch sensation with gloves and socks distribution and weakness at lower limbs associated with moderate muscle hypotrophy. Her body weight was 44 kg (BMI 17.4, mBMI 852). Echocardiography was consistent with amyloid infiltration (mean left ventricular wall (mLVW) thickness 14 mm, ejection fraction [EF] 50%) with N-terminal pro-B-type natriuretic peptide (NT-proBNP) of 2429 pg/mL (u.r.l. 334). Serum and urine immunofixation tests were negative, with free light chains within reference range and a normal κ/λ ratio. An abdominal fat aspirate was positive and genetic testing showed a heterozygous Val30Met *TTR* variant. ATTRv was diagnosed and the patient was started on tafamidis 20 mg/day. Oral suspension erythromycin 250 mg BID was also prescribed, but in spite of an initial benefit for gastric symptoms, it was discontinued because of diarrhoea. Domperidone (10 mg BID before meals) was recommended. Oral nutritional and vitamin D supplementation was also introduced. Vomiting episodes decreased significantly. However, mild morning nausea recurred. Levosulpiride 25 mg twice a day was cyclically alternated with domperidone. Three years after tafamidis was started, her body weight was 50 kg and mBMI remained stable (889).

## Comments

### Pathophysiology of gastrointestinal disturbances

Early satiety, postprandial fullness, nausea, vomiting and weight loss are symptoms of gastroparesis, a delay in the emptying of contents from the stomach into the small bowel in the absence of mechanical gastric outlet obstruction [19]. The pathogenesis of gastric retention and its relationship with GI disturbances is poorly elucidated in ATTRv. In a Swedish series, none of the patients showing a severe delay in gastric emptying on scintigraphy actually complained of upper GI symptoms [2]. In another study from the same centre, gastric emptying appeared stable 5 years after liver transplantation, although the reported symptom scores significantly worsened over time [3].

Published data on gastrointestinal function in ATTRv are largely limited to patients with the V30M variant, but findings are likely representative of other mutations as well. Gastroparesis is generally attributed to autonomic dysfunction, but the actual role of the efferent sympathetic and parasympathetic autonomic system is not clear. An autopsy study revealed pronounced amyloid infiltration and destruction of the vagal nerve and ganglion [20], and a study of oesophageal function noted motility disturbances that were attributable to vagal dysfunction [21]. However, in a study of gastric emptying, the relationship with vagal activity, measured by heart rate variability (HRV), was poor [2].

The enteric nervous system has been examined in autopsy samples, and the enteric nerves and ganglions were surprisingly well preserved in a study of autopsy material from Japan, where amyloid was found in only 18% of the examined ganglia of the myenteric plexus of the stomach. In the small intestine, 6% of examined ganglia showed amyloid deposits and no deposits were found in the myenteric plexus of the colon. However, a reduction in colonic vasoactive intestinal polypeptide (VIP)-immunoreactive nerve fibres and neurons in the myenteric plexus was noted that might be one of the factors contributing to ATTRv patients GI motility disturbances [22, 23]. In another study, degeneration of enteric nerve plexuses was observed in two patients, and heavier amyloid deposition was found in the wall of the stomach than in the rectum [18].

The intestinal endocrine cells have been investigated in the small and large intestine, and profound depletion was noted [24–26]. However, after liver transplantation, the number of endocrine cells returned to normal levels, without a corresponding normalisation of the GI symptoms; thus, the role of endocrine GI cells is uncertain [27]. A recent investigation of the intestinal cells of Cajal in the gastric ventricle noted a marked depletion [28]. This finding is interesting, since a similar depletion has been noted in patients with diabetes mellitus. The cells of Cajal are regarded as the pacemaker cells of the intestine that ensure a normal synchronized motility pattern throughout the intestine. The finding is supported by a study of small intestinal motility, where abnormally configured or propagated phase III complexes (fasting motility pattern) were found in 58% of the patients [1]. The fasting motility pattern ensures clearance of the small intestine after a meal, and abnormal small intestinal motility predisposes to bacterial contamination of the small intestine and also gastric retention. Therefore, gastroparesis in ATTRv patients probably reflects a generally disturbed GI motility pattern, and not merely impaired gastric emptying.

### Diagnosis and monitoring of upper GI dysfunction

Even though the mechanisms leading to GI disturbances are not settled, investigations of upper GI symptoms are warranted in ATTRv patients, together with thorough exclusion of other treatable and reversible causes. Examinations may include endoscopy, oesophageal and intestinal manometry, and gastric emptying studies. Endoscopy is commonly employed in the evaluation of patients with GI disturbances. However, its role in the context of ATTRv is limited. Apart from gastric retention suggested by the presence of residual gastric content after an overnight fast, findings are usually non-specific, including a fine granular appearance of the mucosa, and therefore give no clue to the diagnosis [29]. Biopsy specimens may however be important in confirming the diagnosis by the finding of amyloid deposits. As discussed above, submucosal vessels should be present in the biopsy specimens, since amyloid deposits are rarely found in the mucosa [16].

A high frequency of abnormal oesophageal motility, even in asymptomatic patients, has been reported in a small series [21]. However, the role of oesophageal, as well as intestinal motility investigations in the evaluation of ATTRv patients GI disturbances remains to be established [1]. Apart from endoscopic diagnosis of gastric retention, gastric emptying studies by radioactive labelled meal has been used. In 39% of 162 patients with ATTRv delayed gastric emptying was noted. The correlation between delayed gastric emptying and symptoms was poor, and mostly noted in patients with symptoms from both the upper and lower GI tract [2]. However, gastroparesis significantly correlated with a low mBMI and patients with severe gastric retention had significantly lower mBMI.

Measurement of gastrointestinal motility and transit times, including gastric emptying at “one go”, is now possible with the wireless motility capsule. This is a convenient method that will provide important information, and the device is approved within both the USA and the EU [30]. No study on ATTRv patients has, however, been published.

Finally, in the advanced stages of the disease, adrenal insufficiency secondary to amyloid infiltration may potentially contribute to nausea, weight loss and vomiting. It is thus appropriate to perform a cortisol determination in the morning and, when abnormal, an adrenocorticotropic hormone (ACTH) test [4].

Nutritional status and patients’ reported symptoms are validated tools for defining gastrointestinal involvement and monitoring disease progression both in routine practice and in clinical trials [31, 32]. Commonly used questionnaires developed for assessing upper GI symptoms in several conditions, i.e. the Patient Assessment of Gastrointestinal Disorders Symptom Severity Index (PAGI-SYM) and Gastroparesis Cardinal Symptom Index (GCSI), have not been validated in ATTRv [33]. The Swedish group has proposed a specific questionnaire that proved useful for measuring the extent of GI involvement in several studies [4]. The compound autonomic dysfunction test (CADT), developed in France more than a decade ago, is another simple and reproducible scale that scores upper and lower GI symptoms according to their frequency [34]. The Composite Autonomic Symptom Score (COMPASS)-31 questionnaire was recently used in the registration trial for the siRNA patisiran to account for GI manifestations in combination with other autonomic domains [35].

The mBMI index is a well-established measure of nutritional status that has been shown to correlate with neurological function, including familial amyloid polyneuropathy (FAP) stages, and duration of gastrointestinal manifestations [36]. It deteriorates significantly over time in untreated patients and strongly predicts survival after liver transplantation. It has proved to be an important secondary endpoint in clinical trials for ATTRv, where preservation of mBMI was closely associated with neurological response and improvement in quality of life [37–40].

### Management of symptoms

Treatment should aim at relieving symptoms and halting the progression of gastrointestinal dysfunction in order to preserve nutritional status and quality of life. The therapeutic strategy therefore relies on a combination of specific therapy for ATTRv and supportive care. Effective disease-modifying treatments for hereditary TTR amyloidosis that target different steps of the amyloidogenic pathway are increasingly available [41]. Pharmacological agents include small-molecule TTR stabilizers, namely tafamidis and diflunisal, that prevent tetramer dissociation and protein misfolding, and gene-silencing drugs that, by selectively degrading TTR mRNA into hepatocytes, suppress the synthesis of the circulating precursor [37–40].

Supportive therapy is of outmost importance and should be guided by an accurate evaluation of the patient’s more debilitating symptoms. As in diabetic enteropathy, symptomatic relief of upper GI symptoms is challenging. Moreover, studies in patients with hereditary ATTR amyloidosis are lacking. Table 2 reports conventional symptomatic treatments for upper and lower GI disturbances.

**Table 2:** Medical treatment for gastrointestinal complications in ATTRv

Underlying pathology	Symptoms	Available treatments
Gastroparesis	Early satiety, nausea and vomiting	D2 receptor antagonists
		Domperidone^a^
		Metoclopramide
		Levosulpiride^a^
		Motilin receptor agonists
		Erythromycin
		5-HT4 agonists
		Prucalopride
		Ghrelin agonist
		Relamorelin^b^
Small bowel bacterial contamination	Constipation/diarrhoea, diarrhoea	Antibiotics^c^
		Rifaximin
		Metronidazole
		Amoxicillin/clavulanic acid
		Doxycycline
		Probiotics^d^
Slow transit	Constipation	Osmotic active preparations
		Polyethylene glycol
		Picosulphate^a^
		5-HT4 agonists
		Linaclotide
		Lubiprostone^a^
		Prucalopride
Bile acid malabsorption	Diarrhoea	Bile acid sequestrants
		Cholestyramine (preferably in conjunction with a reduced-fat diet)
Rapid GI transit	Diarrhoea	Opioid receptor agonists
		Loperamide
		Somatostatin analogues
		Octreotide

^a^Not available in all countries

^b^Under consideration for approval by the European Medicines Agency

^c^According to local practice; the most effective antibiotic for this indication is not settled

^d^Substantial variation in types of probiotics among different countries; most preparations contain strains of *Lactobacillus*

Dietary changes may be useful, and advice should include small-volume meals with low soluble fibre and fat content. Nutritional supplementation could also be considered early in the disease course. Prokinetics are the mainstay for the management of symptoms related to gastric paresis. Erythromycin, which acts as a motilin receptor agonist, is the first choice in the Swedish centre in Umeå [4] and may be prescribed in a dose of 50–250 mg two to three times a day. The lower dose at the Umeå centre was found to be as effective as the usual dose of 250 mg three times daily, whereas the higher doses typically employed often induce saturation of the receptor with a rapid decline in response, and increase side effects [42, 43]. Domperidone (10 mg twice daily, 15 min before meals) may be effective in reducing early satiety and in preventing nausea and vomiting episodes. In Italy we prefer it to metoclopramide for prolonged treatment, using short courses of metoclopramide IM or IV on acute attacks of recurrent vomiting. In this context, prompt electrolyte and fluid supplementation is also important to prevent the development of metabolic alkalosis and hypotension related to volume depletion, to which patients with ATTRv may be particularly susceptible because of the concomitant use of diuretics for cardiomyopathy. Levosulpiride may also be considered in cases of reduced appetite due to delayed gastric emptying and is a useful alternative, cyclically, to limit the potential drawbacks of prolonged use of domperidone. However, like domperidone, it is not available in all countries. Novel drugs that stimulate gastrointestinal motility, such as ghrelin agonists, have been investigated in diabetic enteropathy. Relamorelin was proven effective and safe versus placebo in reducing symptoms of gastroparesis in diabetic patients [44]. However, it is not currently available, but is under consideration for approval by the European Medicines Agency. Similarly, benefits were obtained with the novel 5-HT4 agonist prucalopride [45]. Given the similarities to the pathophysiology of diabetic enteropathy, these drugs may reasonably be used for treating ATTRv gastroparesis, although it would be important to evaluate their role in a controlled trial. To our knowledge, there is no relevant experience with gastric stimulators in the management of gastroparesis in ATTRv. In addition, pacemaker treatment constitutes a contraindication.

## Case 2: a young man with weight loss and diarrhoea

A 43-year-old man began to experience bowel abnormalities consisting of periods of marked constipation alternating with occasional loose, non-bloody stools of varying volume. Progressive recurrent episodes of diarrhoea then occurred. Gastroenterological evaluations were performed that excluded celiac disease and endocrine and autoimmune disorders. Stool cultures were negative and inflammatory markers were within reference range, including faecal calprotectin. On laboratory tests only mild macrocytosis was documented, with serum vitamin B_12_ slightly reduced. Lactase deficiency was genetically disclosed, but no significant improvement was obtained from dietary changes. He was treated with probiotics and vitamin supplementation. Colonoscopy was performed twice, with no significant findings and no indication to biopsy. A computed tomography of the abdomen was negative. A gastroscopy showed gastroesophageal reflux disease requiring a proton pump inhibitor. Cholestyramine failed to improve bowel symptoms. A trial with beclometasone was also performed, with no significant benefit. Progressive fatigue and significant weight loss occurred over the following 12 months, with daily diarrhoea and profound muscle weakness. Mesalazine 1000 BID was then started, with no substantial changes. When a second-degree relative was diagnosed with transthyretin cardiac amyloidosis, the patient was referred to our centre for further investigation. On evaluation, he complained of severe fatigue, erectile dysfunction, difficulty in swallowing, chronic diarrhoea with 6–7 movements/day and limb weakness. On his past medical history he reported bilateral carpal tunnel syndrome surgically treated at the age of 37 years. Body weight was 61 kg (BMI 21, mBMI 950). No sensory abnormalities were found on neurological examination, but weakness was reduced in the upper limbs and he had difficulty in rising from a kneeling position. Although asymptomatic, orthostatic hypotension was measured. TTR cardiac amyloidosis was diagnosed on echocardiography and by means of DPD scintigraphy, with NT-proBNP of 532 pg/mL (u.r.l. 88). Serum and urinary immunofixation was negative. Nerve conduction studies showed a mild, predominantly axonal, sensorimotor polyneuropathy. Genetic testing identified heterozygosity for the p.Glu89Gln *TTR* variant, establishing the diagnosis of ATTRv. The severe autonomic dysfunction combined with a non-V30M variant did not support indication to combined liver-heart transplantation in spite of a mild, stage I peripheral neuropathy. Considering the marked bowel abnormalities with undigested pills recurrently found in stools, we recommended enrolment in a clinical trial with the novel gene-silencing agent patisiran. Symptomatic treatment was established with loperamide and octreotide (0.05 mg up to three times a day). The latter was however discontinued shortly afterwards due to recurrent hypoglycemia. Rifaximin and probiotics were recommended cyclically.

## Comments

### Diagnosis and monitoring of lower GI dysfunction

Abnormal bowel function, presenting as either constipation, diarrhoea, alternating constipation/diarrhoea or faecal incontinence, may be the inaugural manifestation of ATTRv, particularly in patients with early-onset V30M or certain non-V30M variants [5, 7]. As discussed above, different mechanisms impair the coordinate neural networks responsible for small bowel motility, resulting in delayed GI transit time and ineffective peristalsis [46]. Small intestinal bacterial overgrowth (SIBO) and bile acid malabsorption, commonly associated with abnormal motility, also contribute to worsened symptoms related to neuropathy [4]. On the contrary, no significant association between hereditary ATTR amyloidosis and other GI disorders that may cause diarrhoea, including celiac disease, microscopic colitis or exocrine pancreatic insufficiency, has been reported to date.

Bacterial contamination of the small bowel is indeed a well-recognized complication of ATTRv. It gives rise to either continuous or intermittent diarrhoea and constipation. The diagnosis can be difficult, since sensitivity of the available tests is relatively low. Culture of aspirates from the small intestine can be used, but the procedure is cumbersome, since anaerobic cultures are required. More commonly available is the hydrogen breath test, where the hydrogen content in expired air is measured after ingestion of glucose. Sensitivity of 60% is reported, and some dietary restrictions are required before the test. Bacterial contamination was found by hydrogen breath tests in 50% of 14 patients undergoing evaluation before liver transplantation. Measurement of methane is also recommended [47], given its importance in GI symptoms and because the potential presence of an excess of methanogenic microbes in the gut may significantly impact on hydrogen measurement, as these bacteria utilize H_2_ in the generation of CH_4_. In a small series of seven patient, abnormal 14C-glycocholate breath test results were found in six [48]. This test relies on de-conjugation of bile acids as a marker of bacterial contamination; however, the possibility of concomitant bile acid malabsorption makes the test unreliable [49]. Nevertheless, the few studies published on bacterial contamination of the small bowel indicate that it is common among those with diarrhoea, and since it is treatable by antibiotics, it should be diagnosed and managed.

Various markers of malabsorption have been used in the evaluation of ATTRv. Fat malabsorption measured by faecal fat determination is rarely performed today, but steatorrhoea is a common finding in ATTRv patients with diarrhoea. In fact, it was found in 30 of 47 patients (58%) examined in a Swedish cross-sectional study [50], and in seven of 16 patients under evaluation for liver transplantation [49]. In a study from Switzerland, six of seven ATTRv patients suffering from diarrhoea were found to have steatorrhoea [48]. Malabsorption of vitamin B_12_ and xylose has also been reported in ATTRv patients [48]. Moreover, the presence of bile acid malabsorption has been shown to affect survival after liver transplantation and to be a common complication in patients with diarrhoea [12, 47, 51]. Diagnosis of bile acid and fat malabsorption is important, as the diarrhoea they cause can often be relieved by appropriate treatment and dietary restrictions. In recent years, the 75-selenium homocholic acid taurine (SeHCAT) test has become increasing used worldwide for the diagnosis of bile acid malabsorption, being simple, fast and well-tolerated, and having higher sensitivity and specificity than other methods [52].

### Management of symptoms

Treatment of lower GI disturbances is extremely important to mitigate symptoms and preserve quality of life, particularly work and social activities, and to avoid malnutrition. Obstinate constipation often precedes the appearance of loose stools and faecal incontinence, but may cause significant discomfort. Patients may benefit from osmotic laxatives and polyethylene glycol is usually the most effective option [4]. Picosulphate can be used, usually as droplets once daily, but it is not generally available in Europe. Newer agents such as linaclotide (once daily), lubiprostone, although not generally available in Europe (twice daily) and prucalopride can be reasonably considered when laxatives have failed [53], although there is no reported experience in ATTRv. Increased dietary fibre content is usually poorly effective.

Diarrhoea, continuous or alternating with constipation, should always be promptly treated, although symptomatic management may be disappointing. Bacterial contamination is a common underlying cause for alternating diarrhoea and constipation, and should be tested for, and treatment with antibiotics could be considered even if the test is negative. Treatment of SIBO should be precautionary, i.e. monthly cycles consisting of rifaximin on days 1–7. The antibiotic course can be followed by probiotics, but the efficacy or type of probiotic is not evaluated on this indication. Data from studies in diarrhoea-predominant irritable bowel syndrome (IBS-D) associated with SIBO suggest a beneficial effect of modulation of gut microbiota by probiotics mostly from the *Lactobacillus* and *Bifidobacterium* genera, although multi-strain formulations have been increasingly investigated [54, 55]. The course of antibiotics is repeated after 14–21 days according to recurrence of diarrhoea. Since rifaximin is not absorbed, it appears attractive to use, but other antibiotics can be given according to local experience, including amoxicillin and clavulanic acid, metronidazole or ciprofloxacin. Given that recurrent treatment with broad-spectrum antibiotics is the current approach in SIBO, the risk of disruption of the gut microbiota and occurrence of antibiotic resistance should not be underestimated. Microbial resistance to rifaximin is considered rare and transient due to the high local concentration of the drug and to the absence of horizontal transmission [56, 57]; however, which agent actually has the most favourable long-term profile remains elusive.

Since bile acid malabsorption and steatorrhoea often are associated with more chronic diarrhoea, a reduced-fat diet together with bile acid sequestrants should be tried, preferably after testing for bile acid malabsorption. Moreover, it is important that the diet is well balanced and sufficient in calories.

Symptomatic treatment with anti-diarrhoeal opioids, i.e. loperamide, is a mainstay of therapy for diarrhoea. Loperamide is recommended on demand, according to the number of daily bowel movements, and doses up to 12–16 mg/day may be allowed [4]. Some patients benefit from regular use of 2 mg of loperamide in the morning and/or in the evening, preventing frequent bowel movements and loose stools.

The management of patients who present with prolonged, severe constipation that alternates with days of diarrhoea, or who develop substantial diarrhoea following the use of even low-dose laxatives, appears particularly challenging. In these cases, a stepwise approach is recommended, i.e. gradually increasing the dosing of laxatives, avoiding their daily use, assessing the tolerability of different osmotic agents, or considering stimulant laxative suppositories to minimize diarrhoea. In most of these cases, bacterial contamination is likely to contribute to the diarrhoeal episodes that alternate with constipation, suggesting that a short cycle of rifaximin when bowel movements increase could also be effective and better tolerated than anti-diarrhoeal opioids, which might worsen the slow-transit constipation.

Somatostatin analogues, particularly octreotide, can be administered to patients with chronic diarrhoea who fail to respond to loperamide. A recent retrospective series in French ATTRv patients refractory to loperamide showed that long-term treatment with octreotide was associated with remission of diarrhoea in two-thirds of cases, improvement in the number of daily movements and reduced faecal incontinence [58]. However, hypoglycemia was the most common side effect, leading to treatment discontinuation in two cases [58]. Although long-acting formulations may be considered, it is wise to start with subcutaneous daily injections (0.05 mg twice a day and slowly increasing up to 0.1 mg three times a day) to allow prompt discontinuation should abdominal pain or hypoglycemia occur.

Vitamin (including vitamins D, A and B_12_) and calcium supplementation are also recommended when diarrhoea becomes persistent. In selected cases, when severe malnutrition develops and/or patients’ quality of life is completely devastated by the high frequency of daily movements usually associated with faecal incontinence, parenteral nutrition may be considered to improve nutritional status while reducing or replacing oral nutrition to control diarrhoea [59]. Diarrhoea is closely related to faecal incontinence, and if the treatment of diarrhoea fails, the remaining option is a stoma. A well-functioning stoma gives patients a chance to control their defecation that otherwise leaves them housebound and isolated. Impaired hand function is a problem, but most patients are able to manage a modern stoma even with impaired hand function [60].

## Conclusions

Early recognition of gastrointestinal dysfunction is important to enable prompt treatment to relieve symptoms, preserve quality of life and prevent malnutrition. Diagnostic tools for GI abnormalities are still limited, and most investigations are not routinely applied in clinical practice. Nutritional status and self-reported symptoms, scored according to validated questionnaires, are presently widely accepted measures for monitoring GI involvement in ATTRv. Better knowledge of the mechanisms responsible for the gastrointestinal manifestations of the disease can provide novel targets for more effective supportive treatment of these symptoms. As in diabetic enteropathy, future developments may include restoration of the gut microbiota or tissue regeneration. These strategies will complement our increasing ability to effectively treat this condition. Indeed, pharmacological approaches are now available that stabilize TTR native structure or suppress its hepatic production, preventing additional amyloid deposition. Gastroenterologists therefore play an important role in ATTRv, and their expertise should be valued for an effective multidisciplinary approach to this disease.
